# Revolutionizing Early Disease Detection: A High-Accuracy 4D CNN Model for Type 2 Diabetes Screening in Oman

**DOI:** 10.3390/bioengineering10121420

**Published:** 2023-12-14

**Authors:** Khoula Al Sadi, Wamadeva Balachandran

**Affiliations:** 1 Department of Electronic and Electrical Engineering Research, Brunel University London, Uxbridge UB8 3PH, UK; wamadeva.balachandran@brunel.ac.uk; 2 Information Technology Department, University of Technology and Applied Sciences-Al-Mussanha, P.O. Box 13, Muladdah 314, Sultanate of Oman

**Keywords:** deep learning, convolutional neural networks (CNNs), k-nearest neighbours (KNN), diabetes type II

## Abstract

The surge of diabetes poses a significant global health challenge, particularly in Oman and the Middle East. Early detection of diabetes is crucial for proactive intervention and improved patient outcomes. This research leverages the power of machine learning, specifically Convolutional Neural Networks (CNNs), to develop an innovative 4D CNN model dedicated to early diabetes prediction. A region-specific dataset from Oman is utilized to enhance health outcomes for individuals at risk of developing diabetes. The proposed model showcases remarkable accuracy, achieving an average accuracy of 98.49% to 99.17% across various epochs. Additionally, it demonstrates excellent F1 scores, recall, and sensitivity, highlighting its ability to identify true positive cases. The findings contribute to the ongoing effort to combat diabetes and pave the way for future research in using deep learning for early disease detection and proactive healthcare.

## 1. Introduction

Diabetes, a chronic metabolic condition characterised by persistent hyperglycaemia, is becoming a major global health concern. This condition profoundly impacts societies and healthcare systems around the globe, causing both economic and societal disruptions [1,2]. The situation is particularly critical in Oman and the Middle East at large, where the prevalence of diabetes has shown an alarming increase, leading to significant socioeconomic burdens [3]. The importance of early diabetes detection is well established, as this condition often goes unnoticed until complications develop, underscoring the need for proactive detection and early intervention. Traditional diagnostic methods for diabetes, such as fasting plasma glucose, oral glucose tolerance tests, and haemoglobin A1c tests, are reliant on the symptomatic manifestation, typically presenting in the disease’s more severe stages [1].

The recent breakthroughs in machine learning and deep learning offer a transformative approach to medical prognosis and diagnosis, unlocking unprecedented prospects for disease prediction, including diabetes. Among the novel technologies, Convolutional Neural Network (CNN), a subset of deep learning algorithms, have displayed significant efficacy. CNNs, along with other machine learning models, can process and analyse extensive medical data, identifying intricate patterns and correlations that can be challenging for human clinicians to perceive. These models can potentially anticipate early signs of diabetes, possibly leading to earlier diagnosis, treatment, and enhanced patient outcomes [1,2,3].

With the promising potential of machine learning in diagnosing diabetes, this research aims to put forth an innovative Convolutional Neural Network model architecture dedicated to early diabetes prediction. The model will make use of a newly collected clinical dataset from Oman, aspiring to achieve high accuracy in predicting type 2 diabetes. By focusing on a region-specific dataset, the study intends to enhance health outcomes for those at risk of developing diabetes in Oman and the wider Middle East [4,5,6].

This research aims to contribute significantly to the worldwide effort to fight diabetes through thorough model development, validation, and performance optimisation. The findings can potentially affect healthcare providers, policymakers, and researchers, with the goal of strengthening early detection strategies and reducing the severe health implications of late-stage diabetes. In the end, the newly proposed CNN model can be a promising tool for diagnosing diabetes, offering critical insights for personalised and proactive diabetes management.

## 2. Related Study Overview: CNNs in Disease Prediction

Convolutional Neural Networks (CNNs) have become a pivotal tool in disease prediction, especially in the realm of diabetes, propelled by the advancements in machine learning and deep learning technologies. Primarily recognised for their prowess in image recognition, CNNs’ application in health informatics has witnessed exponential growth [7,8].

Studies indicate their promising efficacy. For instance, one study demonstrated CNNs’ superior capability in forecasting diabetes remission post-gastric bypass surgery compared to conventional indices [9]. Another used ResNet CNN models in conjunction with numerical data and images, registering prediction accuracies ranging from 77.37% to 90.04% on the PIMA Indian dataset [10]. In another intriguing approach, a hybrid of CNNs and Long Short-Term Memory (LSTM) models showcased high accuracy in diabetes prediction [11], though the suggestion was to further integrate diverse classifiers for enhanced outcomes.

Beyond sheer prediction, CNNs also exhibited proficiency in forecasting blood glucose levels [12]. In a comparative analysis, a CNN model outshone LSTM models in this regard [13]. Yet, challenges arise in long-term predictions, emphasizing the necessity for expansive datasets and effective missing data management strategies. Ongoing studies are focusing on harnessing different activation functions with CNNs for potential optimisation in diabetes predictions [14].

In broader epidemiological scenarios, CNNs have demonstrated aptitude in predicting diseases like influenza-like illness (ILI) [15] and even in classifications within imbalanced datasets with missing values, as observed in a diabetes mellitus study that utilized a Deep 1D-Convolutional Neural Network (DCNN) [16]. Their versatility is further evinced in applications such as heart disease predictions [17,18] and in diagnostic processes for ailments like COVID-19 using medical imaging [18].

Their relevance is not just confined to disease diagnosis. Remarkably, CNNs have ventured into environmental health sectors, exhibiting promise in water quality monitoring by interpreting 2D fluorescence spectra [19].

In sum, CNNs have undeniably marked their presence across diverse applications ranging from computer vision to medical image analysis [20]. Their promise in disease prediction, most notably diabetes, is evident. Yet, the journey towards harnessing their full potential mandates further rigorous research, aiming for refinement and adaptability to ensure maximal contributions to the healthcare sector.

## 3. Materials and Methods

The methodology followed in this study is a systematic sequence of events designed to predict diabetes using Convolutional Neural Network (CNN). A specific dataset from Oman has been utilized to train, validate, and test the model. The methodology includes steps such as loading and pre-processing the dataset and designing a custom 4D CNN architecture.

### 3.1. Dataset

**Figure 1:**
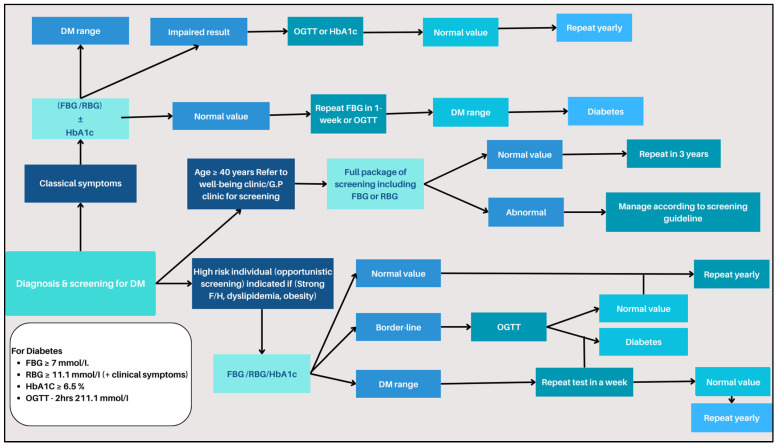
Diabetes screening system workflow.

The dataset pivotal to this research was meticulously assembled, validated, and prepared using diabetes-related data in Oman, in adherence to strict ethical guidelines [21]. The process of diabetes screening system workflow is vividly illustrated in Figure 1.

#### 3.1.1. Data Collection Process

Our data collection procedure involved an extensive collaboration with local diabetes experts and securing necessary approvals from the Ministry of Health, specific health departments, and participating Regional Directorates of Health [21]. The data, drawn from 41 healthcare institutions—including 34 primary healthcare centres, 3 secondary care Extended Health Centres, and 4 local hospitals—highlights the expansive and in-depth nature of our research [22,23].

#### 3.1.2. Inclusion and Exclusion Criteria

The initial pool consisted of potential subjects, all above the age of 20, as per the recent inclusion criteria, even though standard screenings are more prevalent in individuals over the age of 40. The subjects underwent a detailed screening process based on predetermined inclusion and exclusion criteria, ensuring the dataset’s integrity and relevance for our study.

a. Inclusion Criteria:
  - Aged 20 years and above.
  - No prior diabetes diagnosis.
  - Unscreened for diabetes in the past 3 years
b. Exclusion Criteria:
  - Individuals with certain pre-existing conditions (as detailed in Table 1).
  - Those already screened for diabetes at other health centres within the last 3 years.

These stringent criteria ensured the dataset’s robustness, accuracy, and relevance in exploring influential factors affecting diabetes outcomes in the Omani population [21,22].

#### 3.1.3. Data Validation Process

The Al Shifa System, a healthcare information system prevalent in Oman, was critical in our data validation process [23]. Accessible across over 200 healthcare institutions [24], it played a vital role in the rigorous validation of our manually collected data. The system served as a crucial reference point, validating each patient’s clinical results, and filled potential data gaps, enhancing the dataset’s comprehensiveness [23,25].

#### 3.1.4. Dataset Composition and Feature Selection

Our final dataset encompassed 13,224 records, spanning 13 pivotal variables such as age, weight, height, BMI, and more, as illustrated in Table 1. These records were digitally formatted and loaded into MATLAB version 2023b software. The feature selection process was particularly stringent, guided by the criteria defined by Oman’s Ministry of Health for diabetes diagnosis. These features, selected under the supervision of an expert diabetes physician, included factors crucial to diagnosing and managing diabetes [26].

#### 3.1.5. Dataset Utilization and Analysis

For detailed analysis, we converted categorical data into numeric form, which proved essential for various types of analyses, as shown in Table 2. The selected features and subsequent analyses provide an exhaustive insight into the determinants influencing diabetes outcomes, enabling a deeper understanding of the patient demographic and highlighting gaps in existing research [21,26].

**Figure 2:**
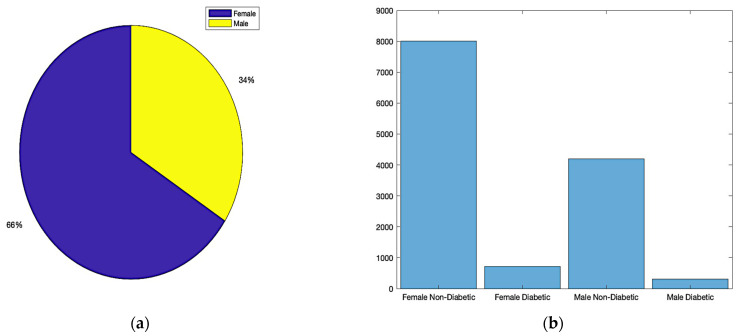
Dataset distribution: (**a**) total gender ratio; (**b**) diabetes status based on gender.

Figure 2 provides a visual summary of our dataset distribution by gender, which is crucial for understanding the gender-wise prevalence of diabetes in the studied population.

The dataset, curated and finalized after a rigorous process of validation and screening, is comprehensive and reliable for the study’s objectives. The careful selection of participants, adherence to the inclusion and exclusion criteria, and the utilization of a robust data validation system ensure the dataset’s accuracy and relevance. This approach not only contributes to the current research on diabetes in Oman but also sets a methodological standard for future studies in similar contexts. The comprehensive nature of these data, starting from individuals aged 20, highlights the study’s thoroughness and its potential to guide future preventive and clinical strategies for diabetes management [21,26].

### 3.2. Exploratory Data Analysis (EDA) 

Visualizing data is paramount in exploratory data analysis. It gives insights into data distribution, relationships between variables, and any potential anomalies. Below, we delve into different visualization techniques applied to our dataset.

#### 3.2.1. Statistical Summary

**Figure 3:**
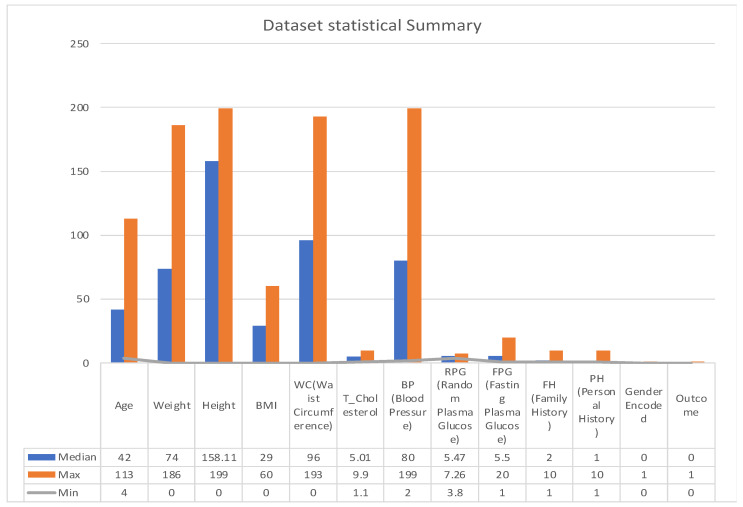
Statistical summary.

**Figure 4:**
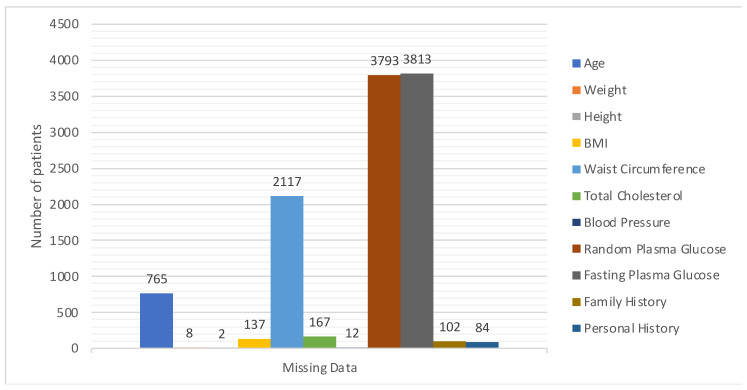
Details of missing values.

A statistical summary provides an insight into the key characteristics of each variable in the dataset. This summary encompasses range, central tendencies (like median), and any potential missing values. The dataset under examination, as summarized in Figure 3 and Figure 4, offers a comprehensive collection of health metrics.

This spans from general health indicators like age (with a range from 4 to 113 years and a median of 43) and weight (ranging between 0 and 186 with a median at 74) to BMI, which has a median of 29, albeit with 137 missing values. Further diving into specialized health markers, we have measurements like random plasma glucose, which interestingly has 3793 missing data points, and a median value of 5.47.

Waist circumference and total cholesterol also contribute to the dataset’s breadth, with respective medians of 95.354 and 5.01. Furthermore, the dataset comprises data on blood pressure, with values spanning from 2 to 199 and a median of 80. However, it is essential to note that 12 values in this variable are missing.

The dataset also integrates personal and family medical histories, each with its own set of missing data (84 and 102 missing values, respectively), suggesting that some patients might not have disclosed or had access to this information. In terms of gender distribution, the dataset employs an encoding mechanism, with 0 representing males and 1 representing females. Finally, the ‘Outcome’ variable, presumably indicating the result or diagnosis, categorizes data into either 0 or 1, though the specifics of these categories were not provided in the summary [27]. 

One key observation from Figure 4 is the presence of missing data across various variables. This can potentially impact the accuracy and reliability of any predictive modelling drawn from this dataset. Handling such gaps, through techniques like imputation, becomes pivotal to ensure robust data analysis. The extensive range observed in variables such as ‘Age’ and ‘Blood Pressure’ underscores the diverse patient cohort represented in this dataset, which is advantageous for establishing a comprehensive and inclusive predictive model.

#### 3.2.2. Histograms

**Figure 5:**
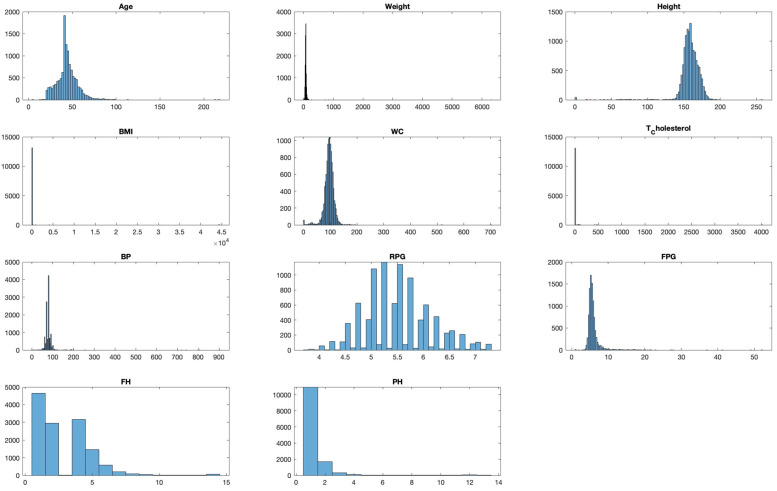
Distribution analysis of each feature in the dataset.

Histograms divide data into bins and visualize the frequency of observations within each bin, helping identify the shape of the data distribution. For example, a histogram for ‘Age’ might reveal a larger number of younger patients compared to older ones, which could be important for the subsequent modelling phase. As presented in Figure 5, we visualize the distribution of each variable to understand their spread and identify any potential outliers.

#### 3.2.3. Scatter Plots

Scatter plots are foundational in visualising relationships between variables. In cases where we want to examine the relationship across three metrics, a 3D scatter plot is employed. By plotting ‘Age’, ‘Weight’, and ‘Height’ on a 3D plane, we can uncover the clusters of data points that share similar characteristics, the potential outliers that deviate from expected trends, and the interactions between the variables that might not be evident in two-dimensional plots.

**Figure 6:**
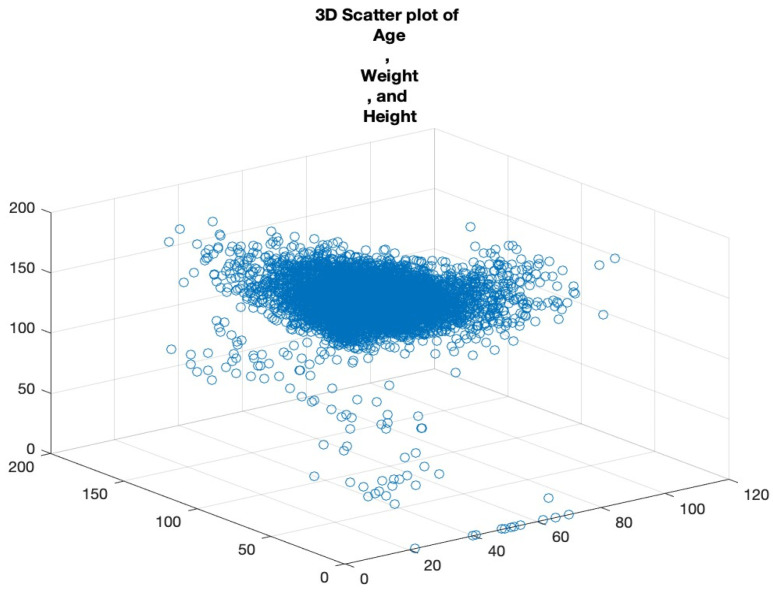
Three-dimensional scatter plot of age, weight, and height.

Rotating and examining this plot from multiple perspectives allows for a more comprehensive understanding of the variables’ relationships. See Figure 6.

#### 3.2.4. Correlation Matrix

**Figure 7:**
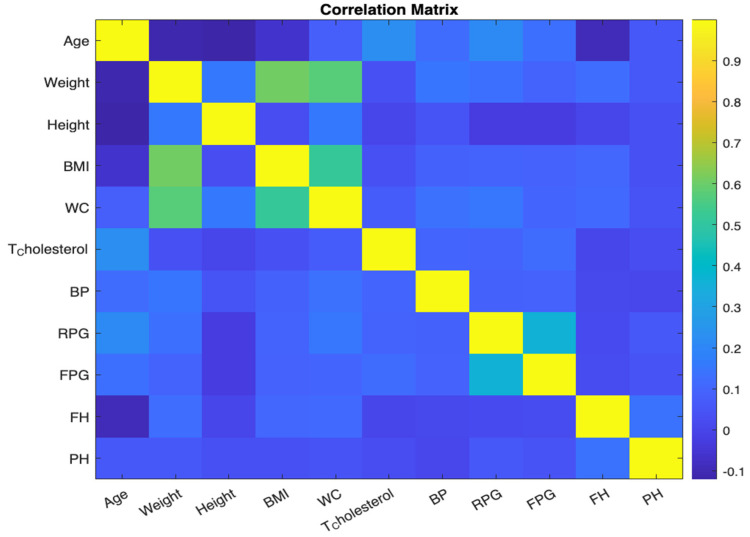
Correlation matrix.

Correlation offers insights into the relationship between variables. We computed a correlation matrix for our dataset to understand the pairwise association of columns. This matrix, visualized using a color-coded grid, indicates the correlation strength and direction between pairs of variables. Highly correlated features may be indicative of redundant information, vital when choosing features for model building. See Figure 7. Each cell in the grid corresponds to a pair of variables, and the colour of the cell represents the strength and direction of the correlation between those variables. The x and y axes are labelled with the variable names for clarity. By examining the colour of each cell, we can quickly identify pairs of variables that are strongly correlated.

#### 3.2.5. Bar Charts

**Figure 8:**
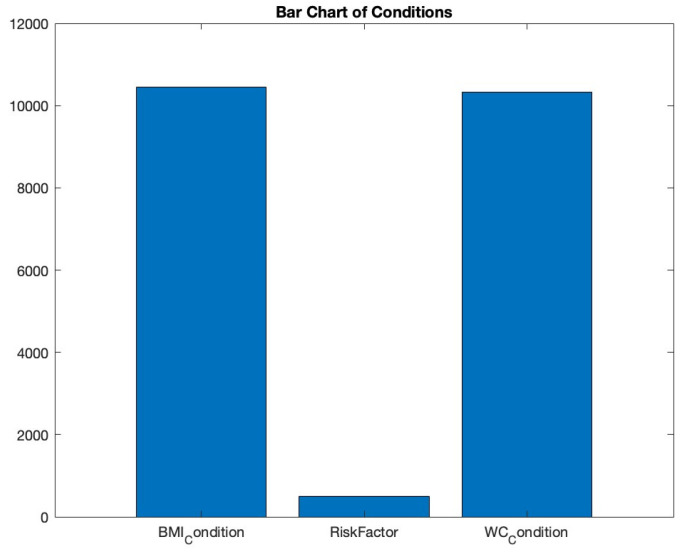
Bar chart of conditions.

Bar charts effectively visualize categorical data by using rectangular bars to depict category frequency. To understand the prevalence of various health conditions, we employed a bar chart in Figure 8. By aggregating the count of conditions like ‘RiskFactor’, ‘BMI_Condition’, and ‘WC_Condition’, the resulting chart offers a concise visual depiction of condition distribution. This helps in recognizing dominant conditions in the dataset.

### 3.3. Pre-Processing the Dataset for CNN Model Training

#### 3.3.1. Data Cleaning and Limit Application

Our pre-processing commenced by focusing on key metrics such as “Age”, “Weight”, and “Height”. We established upper thresholds for each of these, grounded in domain knowledge. For instance, an age beyond 120 years would be regarded as an outlier. Data exceeding these set limits were flagged and effectively labelled as unavailable or ‘NaN’. 

#### 3.3.2. Addressing Missing Data

Missing data are a persistent challenge in real-world datasets, and our collection was no exception. We used the ‘ismissing’ function to detect these absences, yielding a logical map pinpointing the gaps. Each column’s data voids were subsequently summarized and logged for reference (See Table 2). A systematic examination allowed us to identify and index these absences, with a comprehensive summary of our findings presented in Table 2.

To tackle this issue, the K-Nearest Neighbours (KNN) method was chosen. The MATLAB’s ‘fillmissing’ function, paired with the ‘KNN’ parameter, served our purpose, fortifying the data’s internal structure and ensuring analytical veracity. The KNN algorithm estimates missing values by comparing them to similar records in the dataset. This is especially effective when data exhibit strong patterns or correlations between variables [28,29]. For example, if one were missing the weight data for a particular entry but knew the height and age, the KNN method would find other records with similar height and age and use their weight data to estimate the missing value [30,31].

Take, for instance, a missing value in the “Weight” column for an individual aged 25. Leveraging KNN, the system would reference weights of other 25-year-olds within the dataset, determining a plausible estimate grounded in this comparative context. This methodology truly shines when data are characterized by discernible patterns or notable correlations between variables [32]. It not only preserves, but enhances, the inherent structure and relationships within the dataset, ensuring analyses and predictive modelling are both accurate and reliable [32,33].

#### 3.3.3. Removing Outliers with the Z-Score Method

Outliers can distort analyses, leading to potentially misleading conclusions. We turned to the Z-score method for the effective identification and removal of these anomalies [34]. Z-scores represent how many standard deviations a data point is from the mean. For instance, a Z-score of 2 indicates the data point is two standard deviations above the average.

We decided that data points with an absolute Z-score greater than 3 were outliers. This threshold is standard in many domains, ensuring data within a reasonable range of deviation are retained. Once outliers were identified, they were flagged and then addressed using the previously mentioned KNN method to preserve the integrity of the dataset.

#### 3.3.4. Feature Processing

Following data pre-processing, specific clinical features are processed to generate new binary features that aid in predictive accuracy. The following feature processing operations were performed:

- Risk Factor (PH): The attribute “PH” (personal history) was converted into a binary variable indicating whether the value is greater than or equal to 3.
- BMI and Waist Circumference: The attributes “BMI” and “WC” (waist circumference) were converted into binary variables indicating whether the values are above certain thresholds (BMI ≥ 25 kg/m², WC (M) ≥ 94cm, WC (F) ≥ 80cm).
- Mean Blood Pressure: The attribute “BP” (blood pressure) was converted into a binary variable indicating whether the value is greater than or equal to 85 mmHg diastolic.
- Abnormal Blood Sugar: The attributes “FPG” (fasting plasma glucose) and “RPG” (random plasma glucose) were converted into a binary variable indicating whether the values fall within specific ranges (5.6 ≤ FPG < 7 or 5.5 ≤ RPG < 11.1).
- Cholesterol: The attribute “T_Cholesterol” (total cholesterol) was converted into a binary variable indicating whether the value is greater than or equal to 5.2 mmol/L.

#### 3.3.5. Target Variable Encoding

The target variable “Outcome” was initially categorical. To enable training the CNN model, it was converted into numeric labels using the grp2idx function.

#### 3.3.6. Post-Processing Remarks

**Figure 9:**
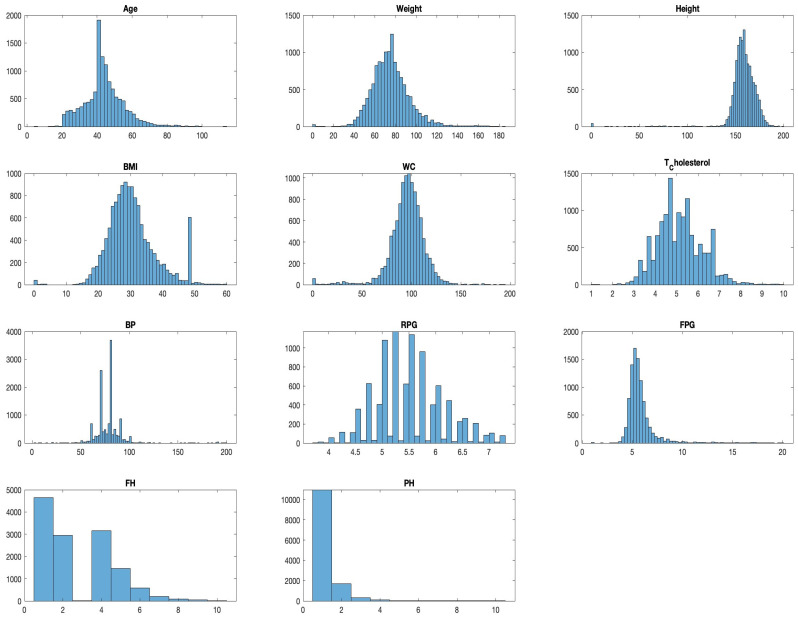
Distribution analysis for dataset after pre-processing.

Through adept application pre-processing approaches, our dataset emerged more realistic and ready for model training. The KNN method ensured missing values were handled judiciously, retaining the inherent relationships in the data. Concurrently, the Z-score method was instrumental in identifying and mitigating anomalies. The transformed dataset can be visualized in Figure 9, depicting its distribution following these pre-processing efforts.

### 3.4. Novel 4D CNN Model for Diabetes Prediction

**Figure 10:**
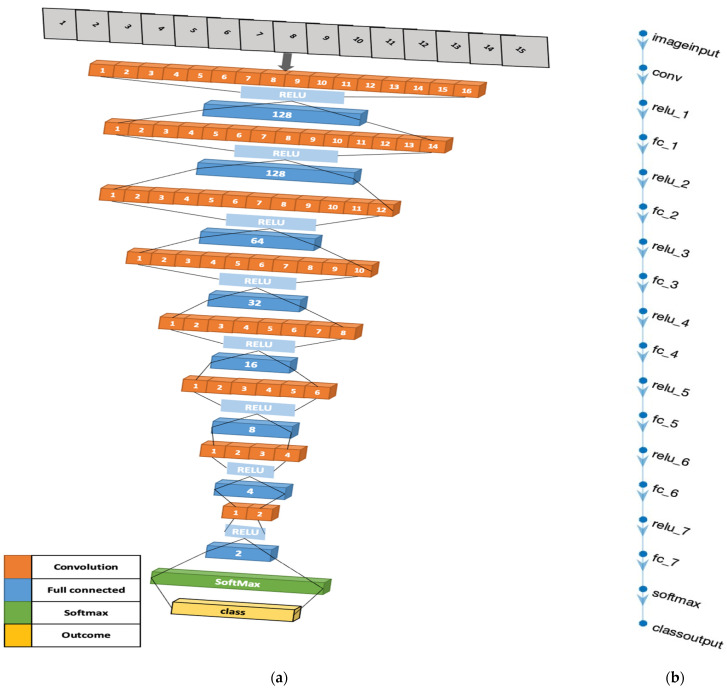
Four-dimensional CNN architecture illustrations: (**a**) The three-dimensional design of the 4D-CNN model architecture. (**b**) MATLAB illustration of the model.

The advent of our 4D Convolutional Neural Network (CNN) model for diabetes prediction marks a significant leap forward in the fusion of machine learning with healthcare analytics. This model diverges from traditional CNN applications by adopting a four-dimensional (4D) input structure, a design that is succinctly illustrated in Figure 10. The “4D” designation refers to an input array with dimensions of [height, width, depth, num_samples], where height and width are minimized to 1, thereby accentuating the depth, indicative of the number of features in the dataset, and num_samples, denoting the dataset’s instance count.

The architectural rationale of our 4D CNN model, as visually depicted in Figure 10, is carefully crafted to balance computational efficiency with the ability to process complex data patterns. The model’s convolutional layers, equipped with [1,1] kernels and 16 filters, perform point-wise convolutions—a technique that is crucial for extracting intricate patterns and interactions within the dataset, vital for discerning signs indicative of diabetes. The fully connected layers, arranged in a descending neuron count (128, 64, 32, 16, 8, 4, 2), simplify the data into a more abstract yet informative representation, crucial for effective diabetes prediction [35,35].

Integral to the model’s design are the Rectified Linear Unit (ReLU) activation functions. These layers introduce necessary non-linearity, enabling the model to adapt to the complex, non-linear relationships found within medical datasets, thus enhancing its predictive accuracy [36]. The architecture culminates with a softmax layer, which computes the probability of each class, followed by a classification layer that assigns each input to the class with the highest probability. This final step is crucial for transforming the processed data into tangible predictions, categorizing each instance into diabetic or non-diabetic classifications [37].

In contrast to other existing models for diabetes prediction that typically employ traditional machine learning approaches [38], utilize Lasso regularization for feature selection [39], or combine CNN with other deep learning models such as Bi-LSTM [34], our model stands out. It utilizes a 4D CNN with a novel architectural design, providing an efficient, effective, and straightforward method for predicting diabetes from complex, multivariate data.

The 4D CNN model’s commitment to CNN methodologies, despite its innovative adaptation to a 4D input structure, is crucial to its capability to handle complex datasets, a common occurrence in medical analytics. Each feature in the input data is treated as a distinct dimension, akin to how an image-based CNN interprets colour channels. This approach enables a comprehensive analysis of the data and an effective extraction of pertinent features, underlining the model’s alignment with traditional CNN principles, yet tailored for non-image data applications like diabetes prediction [40].

The 4D CNN model exemplifies a significant development in diabetes prediction. Leveraging advanced neural network architecture to effectively process intricate multivariate data, its strategic design choices, encompassing both the number and types of layers, are aptly aligned with the complexities inherent in diabetes-related data. This model not only demonstrates the versatility of CNNs in handling diverse data formats but also opens new pathways in predictive analytics for diabetes, highlighting the expansive potential of deep learning technologies in healthcare.

### 3.5. Training and Validation of the Proposed 4D CNN Model 

The training and validation phases for the proposed 4D Convolutional Neural Network (CNN) model involved splitting the dataset into discrete subsets for training, validation, and testing. We employed MATLAB’s inbuilt capabilities to carry out this division, thereby ensuring consistency in results across various runs [41].

For this division, we utilised the ‘cvpartition’ function with a ‘Holdout’ parameter value set at 0.2. This partitioning strategy allows for 20% of the data to be held back for validation and testing purposes, whereas the remaining 80% is utilized for training. This Holdout validation method, originally established by Kohavi in 1995, is a frequently adopted approach in machine learning for model development [42]

The training data (XTrain, YTrain) incorporates features and labels from the primary data (X, Y), respectively. The residual 20% of data is then evenly divided into validation (XValidation, YValidation) and testing (XTest, YTest) sets. It is critical to note that the labels for the validation and testing sets were converted to a categorical format to ensure compatibility with the CNN.

The next step involved reshaping the feature data to match the format required by the CNN model, thereby creating a 4D matrix. This restructuring procedure guarantees that each sample in the training, validation, and testing datasets is perceived as an independent channel.

Our proposed CNN model comprises multiple layers such as 2D convolutional layers, rectified linear unit (ReLU) layers, fully connected layers, a softmax layer, and a final classification layer. The model was constructed using MATLAB’s ‘trainNetwork’ function, which specifies Stochastic Gradient Descent with momentum (‘sgdm’) for model optimization [36].

To determine the optimal number of epochs for model training, we tested a range of values—10, 20, 30, 50, 100, 150, and 200. For each epoch value, the CNN was trained, and the performance was visualized with MATLAB’s built-in plotting functionality. To avoid overfitting and ascertain the best epoch for the model, we employed the validation data (XValidation, YValidation) during the training phase [37].

After the models were trained, they were tested using unseen testing data, which enabled an unbiased evaluation of their performance. This procedure led to the computation of several performance metrics such as accuracy, F1 score, recall, and sensitivity, thus offering a comprehensive understanding of the model’s classification abilities.

The 4D CNN model outlined was trained and validated using a sequence of defined steps. The procedures involved in this process were thorough, ensuring data integrity and reliable outcomes.

## 4. Results of the 4D CNN Model Evaluation

Within the realm of deep learning, our introduced 4D Convolutional Neural Network (4D CNN) model emerges as a sophisticated computational construct tailored specifically for the predictive diagnosis of Type 2 diabetes mellitus (DM). The model was trained, validated, and tested on samples consisting of 10,580, 1322, and 1322 subjects, respectively. One of the primary challenges in the field of medical prediction lies in the careful selection of evaluation metrics. In a typical screening scenario, accuracy might not adequately capture a model’s diagnostic efficacy, especially when a large proportion of the screened population is non-diabetic. A model could, in theory, attain high accuracy merely by predicting most negative outcomes. Recognizing this potential pitfall, our assessment framework pivots on more informative metrics like sensitivity and false referral rates to provide a nuanced understanding of the model’s performance.

### 4.1. Probing the Confusion Matrix

Table 3 offers a granular view of our model’s predictions against actual classifications on the test data.

From the confusion matrix, we extract crucial diagnostic metrics. Sensitivity, which is of paramount importance in a pre-screening context, stands at approximately 90.2%, calculated as:

$$Sensitivity=\frac{True\;Positives}{True\;Positives+False\;Negatives}=\frac{92}{92+10}\approx 90.2\%$$

This high sensitivity indicates that out of all of the diabetic subjects, our model successfully identified about 90.2%. Remarkably, the false referral rate was 0%, computed as

$$False\;Referral\;Rate=\frac{False\;Positives}{True\;Negative+False\;Positives}=\frac{0}{1220+10}=0\%$$

This implies that there were no instances where non-diabetic subjects were incorrectly classified as diabetic, thereby eliminating the risk of unnecessary medical follow-ups and associated expenditures.

### 4.2. Epoch-Driven Performance Analysis 

The effectiveness of a Convolutional Neural Network (CNN), specifically our novel 4D CNN model, is deeply rooted in the number of its training epochs. Each epoch represents a full cycle through the entire training dataset. This section delves into the nuanced impact of epoch variations on the model’s performance, elucidating its learning trajectory and diagnostic precision in the context of Type 2 diabetes mellitus (T2DM). Table 4 below illustrates the performance metrics across diverse epochs, offering a quantitative perspective on the model’s evolving capability.

Accuracy, a ubiquitous metric, measures the proportion of total predictions that the model gets right, considering both positive and negative classifications. While accuracy is undeniably essential, its potential pitfalls, especially in imbalanced datasets, necessitate complementary metrics. Our model’s accuracy showcases consistency across epochs, denoting its consistent performance.

Sensitivity, also termed as the true positive rate, measures the proportion of actual positives (in this case, T2DM diagnoses) correctly identified by the model. This metric is vital, especially when the cost of false negatives (missing an actual positive case) is high, as in disease diagnosis scenarios. Remarkably, our model displays high sensitivity values, underscoring its prowess in timely and correct T2DM detection.

Recall, akin to sensitivity in this binary classification context, emphasizes the importance of capturing as many positive T2DM cases as possible. The model’s impressive recall values further cement its role in T2DM detection.

The F1 score, a harmonized measure of the model’s precision and recall, provides a balance between the two. Consistently high F1 scores across the epochs highlight the model’s sustained efficiency in offering a balanced performance.

**Figure 11:**
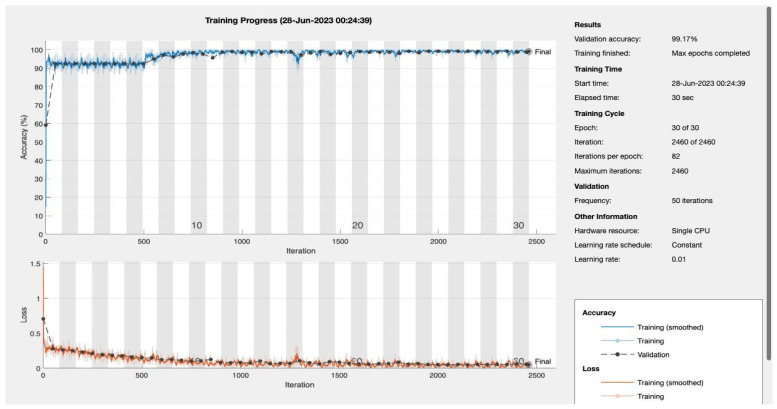
Epoch 30: 99.17% validation accuracy.

**Figure 12:**
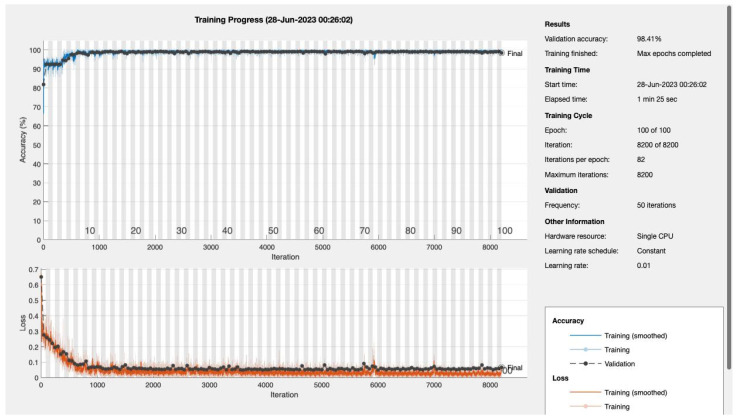
Epoch 100: 99.41% validation accuracy.

Epochs were discerningly selected to represent various stages of model maturation as detailed in Appendix A. Figure 11 and Figure 12 portray the performance trajectory at the 30th and 100th epochs, respectively. These figures, when interpreted alongside Table 2, visualize the model’s learning evolution.

Conclusively, among the 1322 subjects who were screened, our model demonstrated its efficacy by correctly predicting approximately 90.2% (92 out of 102) of the patients diagnosed with T2DM. Furthermore, the model maintained a false referral rate of about 0% (0 out of 1220), showcasing its reliability and precision in identifying T2DM cases without burdening the healthcare system with false positives.

## 5. Discussion

The performance of our proposed 4D CNN model is comparable, if not superior, to that of other state-of-the-art methods for predicting diabetes.

A recent study applied various machine learning algorithms to the Pima Indian Diabetes dataset and achieved an accuracy of 88.6% using a neural network model with two hidden layers [40]. Our CNN model, in contrast, achieved an accuracy well above 98% across all tested epochs, signifying a considerable improvement.

Furthermore, another study developed a convolutional LSTM model for diabetes detection and found it outperformed other models, demonstrating the effectiveness of deep learning techniques in diabetes prediction [43]. While the precise performance metrics were not explicitly reported, our model’s high accuracy and robust F1 score, recall, and sensitivity metrics suggest that it can hold its ground against other high-performing models.

Interestingly, a study comparing different deep learning architectures, including AlexNet, VGG Net, ResNet, DenseNet, and EfficientNet for diabetic retinopathy detection, showed that these models could achieve remarkable results [44]. Although our study differs in the target condition and input data (we focused on general diabetes prediction rather than diabetic retinopathy), our CNN model’s performance is in line with these high-performing architectures, further reinforcing the effectiveness of CNNs in medical prediction tasks.

Finally, our study further confirms the value of machine learning and deep learning techniques for early disease detection, as emphasized in numerous other studies [40,43,44,45,46]. By accurately predicting the presence of diabetes, our proposed model could aid in the early detection and treatment of this prevalent condition, potentially saving lives and reducing the burden on healthcare systems.

## 6. Conclusions

In conclusion, our research presents a ground-breaking approach to diabetes prediction through the development of a novel 4D CNN model. The model’s architecture, specifically designed for multivariate data, demonstrates superior accuracy in early diabetes detection compared to traditional methods. The high performance of the model, as evidenced by impressive metrics such as accuracy, F1 score, recall, and sensitivity, validates its potential as an effective tool for personalized and proactive diabetes management. This research contributes to the global effort in fighting diabetes and holds promise for broader applications of CNNs in disease prediction and healthcare analytics. Implementing our proposed CNN model could have a profound impact on healthcare providers and policymakers.

## Figures and Tables

**Table 1:** Diabetes Screening Eligibility Criteria.

Section	Category/Sub-Category	Details or Criteria
Eligibility for Screening	Diseases Present	If “Yes” to D.M, HTN, CKD: Not eligible for screening.
	Screened in Last 3 Years	If “Yes” to screening at any other health centre in the last 3 years: Not eligible for screening.
Outcome of Screening	-	If “Yes” to any of the above criteria: Excluded from screening. If “No” to both criteria: Proceed to screening.
Family and Personal History	Family History	Obesity, Hypertension, Dyslipidemia, DM, CKD, Premature Cardiovascular Death (M: < 55, F: <65)
	Personal History	Physical inactivity, Ethanol, Tobacco (Cigarettes, Sheesha, Non-smoked tobacco), Nephrotoxic Drugs (NSAIDs, Analgesics, Diuretics, Antibiotics, Herbal)
Reason for Referral to GP	1. Lifestyle Risk Factors	Physical inactivity, smoking, ethanol
	2. Obesity Metrics	BMI ≥ 25 Kg/m² and/or Waist Circumference (M: ≥ 94cm, F: ≥ 80cm)
	3. Blood Pressure	Mean B.P. > 130 mmHg systolic and/or Mean B.P. ≥ 85 mmHg diastolic
	4. Impaired Blood Sugar	FPG (5.6 to < 7.0 mmol/L) or RPG (5.5 to < 11.1 mmol/L)
	5. Diabetes Diagnosis	FPG ≥ 7.0 mmol/L or RPG ≥ 11.1 mmol/L
	6. Cholesterol Level	Serum Cholesterol > 5.2 mmol/L

**Table 2:** Feature selection.

Feature	Description	Data Type
Age	Age of the patient (20–65 years)	Double
Weight	Weight of the patient	Double
Height	Height of the patient	Double
BMI	Body Mass Index	Double
WC	Waist Circumference	Double
T_Cholesterol	Total Cholesterol	Double
BP	Blood Pressure	Double
RPG	Random Plasma Glucose	Double
FPG	Fasting Plasma Glucose	Double
FH	Family History of Diabetes	Double
PH	Personal History of Diabetes	Double
Gender Encoded	Encoded Gender of the patient	Double
Outcome	Diabetic or not	Double

**Table 3:** Confusion Matrix for the Test Data.

	Predicted Non-Diabetic	Predicted Diabetic
Actual: Non-diabetic	1220	0
Actual: Diabetic	10	92

**Table 4:** Epoch-wise Performance Metrics of the 4D CNN Model.

Epochs	Accuracy (%)	F1 Score (%)	Recall (%)	Sensitivity (%)
10	98.487	89.13	80.392	100
20	99.168	94.359	90.196	98.925
30	98.638	90.323	82.353	100
50	98.941	92.929	90.196	95.833
100	99.168	94.359	90.196	98.925
150	99.092	93.878	90.196	97.872
200	98.638	91.000	89.216	92.857

**Figure A1:**
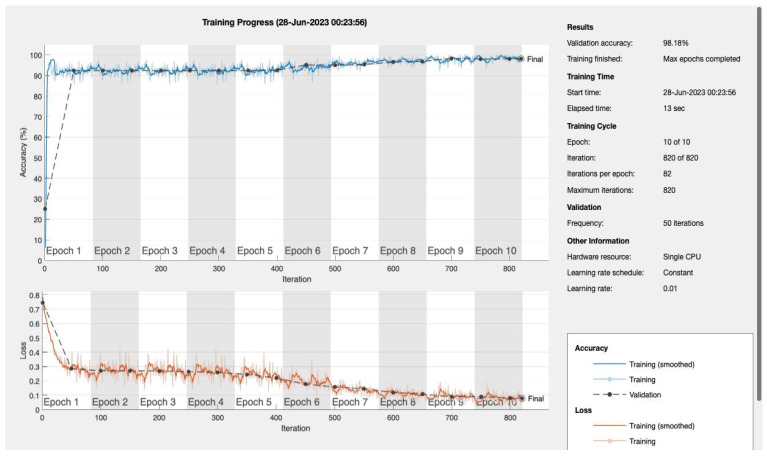
Epoch 10.

**Figure A2:**
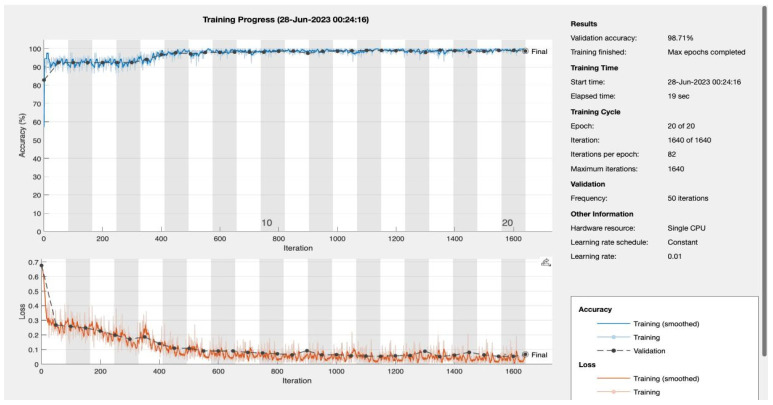
Epoch 20.

**Figure A3:**
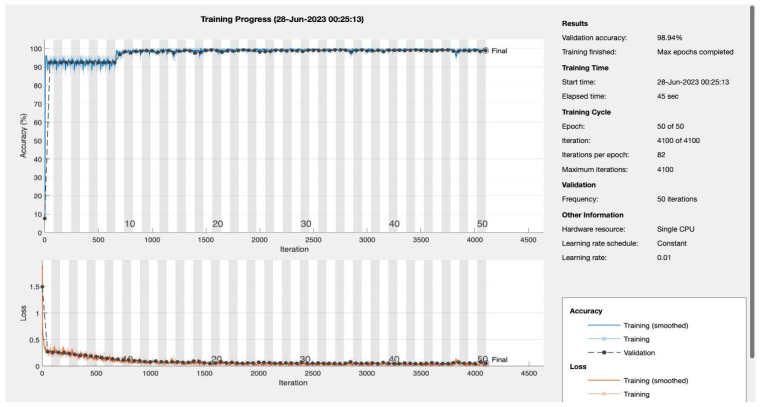
Epoch 50.

**Figure A4:**
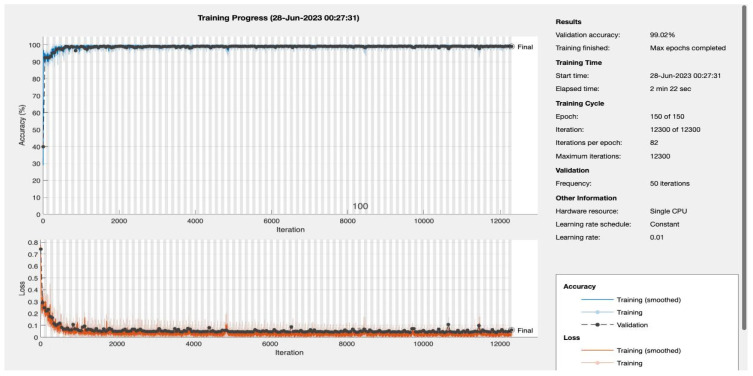
Epoch 150.

**Figure A5:**
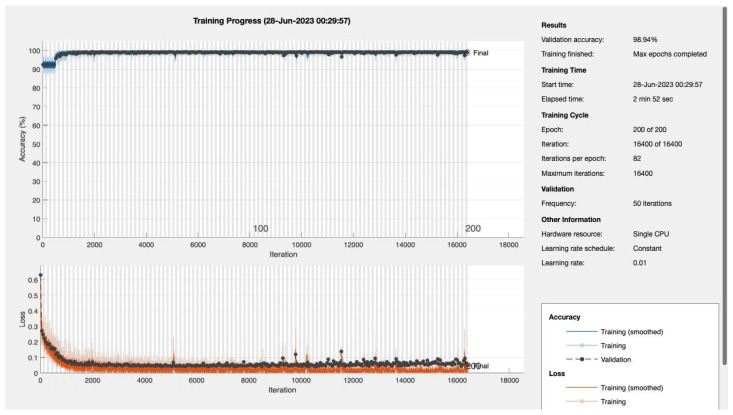
Epoch 200.

## Data Availability

The uniquely constructed Oman Diabetes Type II Screening Dataset, which substantiates the findings of this study, can be made available upon reasonable request by contacting the corresponding author.

## Appendix A

Model Training and Testing: The dataset was split into training, validation, and testing sets using the Holdout method. The CNN model was trained using different numbers of epochs (10, 20, 30, 50, 100, 150, 200). For each epoch value, the model was trained and tested, and the performance metrics were evaluated.
